# Pasireotide in the Personalized Treatment of Acromegaly

**DOI:** 10.3389/fendo.2021.648411

**Published:** 2021-03-16

**Authors:** Manel Puig-Domingo, Ignacio Bernabéu, Antonio Picó, Betina Biagetti, Joan Gil, Cristina Alvarez-Escolá, Mireia Jordà, Montserrat Marques-Pamies, Berta Soldevila, María-Angeles Gálvez, Rosa Cámara, Javier Aller, Cristina Lamas, Mónica Marazuela

**Affiliations:** ^1^ Endocrinology & Nutrition Service, Germans Trias Hospital and Research Institute, Badalona, Autonomous University of Barcelona, Badalona, Spain; ^2^ Endocrinology & Nutrition Service, Complejo Universitario de Santiago de Compostela, Santiago de Compostela, Spain; ^3^ Endocrinology & Nutrition Service, University Hospital, Alicante, Spain; ^4^ Endocrinology & Nutrition Service, Vall d’Hebron University Hospital, Barcelona, Spain; ^5^ Endocrinology & Nutrition Service, La Paz Hospital, Madrid, Spain; ^6^ Endocrinology & Nutrition Service, Reina Sofia University Hospital, Córdoba, Spain; ^7^ Endocrinology & Nutrition Service, La Fe University Hospital, Valencia, Spain; ^8^ Endocrinology & Nutrition Service, Puerta de Hierro University Hospital, Majadahonda, Spain; ^9^ Endocrinology & Nutrition Service, Complejo Hospitalario Universitario de Albacete, Albacete, Spain; ^10^ Endocrinology & Nutrition Service, La Princesa University Hospital, Madrid, Spain

**Keywords:** resistance to medical treatment in acromegaly, somatostatin analogues, somatostatin receptor ligands, personalized medicine, somatotroph adenoma, growth hormone, PitNETs, endocrine tumors

## Abstract

The delay in controlling the disease in patients who do not respond to first-line treatment with first generation somatostatin receptor ligands (first-generation SRLs) can be quantified in years, as every modification in the medical therapy requires some months to be fully evaluated. Considering this, acromegaly treatment should benefit from personalized medicine therapeutic approach by using biomarkers identifying drug response. Pasireotide has been positioned mostly as a compound to be used in first-generation SRLs resistant patients and after surgical failure, but sufficient data are now available to indicate it is a first line therapy for patients with certain characteristics. Pasireotide has been proved to be useful in patients in which hyperintensity T2 MRI signal is shown and in those depicting low *SST2* and high expression of *SST5*, low or mutated *AIP* condition and sparsely granulated immunohistochemical pattern. This combination of clinical and pathological characteristics is unique for certain patients and seems to cluster in the same cases, strongly suggesting an etiopathogenic link. Thus, in this paper we propose to include this clinico-pathologic phenotype in the therapeutic algorithm, which would allow us to use as first line medical treatment those compounds with the highest potential for achieving the fastest control of GH hypersecretion as well as a positive effect upon tumor shrinkage, therefore accelerating the implementation of precision medicine for acromegaly. Moreover, we suggest the development, validation and clinical use of a pasireotide acute test, able to identify patients responsive to pasireotide LAR as the acute octreotide test is able to do for SRLs.

## Introduction

Acromegaly is a heterogeneous disease. Somatotropinomas, the cause of acromegaly in more than 95% of cases (1) as all neuroendocrine tumors, show an intrinsic biologic heterogeneity (2). These tumors present a clinical expression ranging from a small localized microadenoma with limited or controllable biochemical activity to large, invasive, and highly active macroadenomas sometimes poorly responsive to pharmacologic agents acting on the tumor. Thus, this heterogeneity is also reflected in the therapeutic response observed in every single case to the different medical options currently available. Historically, clinical guidelines have not included a personalized approach when recommending the best treatment option for a specific patient (3–5). Most if not all current treatment algorithms for acromegaly are based on a “trial and error” approach which precludes the use of added treatment options when the disease is not controlled with the former prescribed drug (6). In many therapeutic indications -mostly in the cancer field- there has been a conscious move towards personalizing treatment with medication that best matches the characteristics of the disease; however, this trend has not yet fully taken hold in the management of patients with acromegaly.

Due to the current diversity of treatments options, mostly those of pharmacologic nature, it is time to include in medical guidelines for acromegaly, recommendations based on biomarkers that could reliably identify a positive drug response for a given patient.

Lately, different authors have suggested to move on also in the case of medical treatment of acromegaly and set up recommendations for defining algorithms allowing to implement precision medicine in acromegaly patients (7–11).

Regarding medical treatment of acromegaly, different imaging (12) and functional tests performed at the time of diagnosis, before surgical treatment (13, 14), and relevant information obtained from the pathological sample tumor when the patient is operated (15, 16) could be useful to include as predictor biomarkers in the therapeutic algorithm, suggesting that we are currently much closer to personalized treatment for acromegaly. The implantation of a treatment algorithm based upon the prognostic and predicate value of specific biomarkers identifying the response to a given drug has the potential to be more efficacious and cost-effective in the long run.

## Current Options of Pharmacologic Treatment of Acromegaly

The general aim of therapy in acromegaly is to suppress hypersecretion of GH and IGF-1, consequently eliminating morbidity and reducing mortality rates (17). When pharmacologic treatment is to be used, there are currently different compounds including dopamine agonists, first-generation somatostatin receptor ligands (first-generation SRLs), second-generation somatostatin analogs such as pasireotide, the GH receptor antagonist pegvisomant, and others as antisense oligonucleotide drugs (18), these latter currently in clinical development, but potentially available in the years to come.

In the mid-’70s it was discovered that dopaminergic stimulation, contrary to what happens in physiological conditions, reduced GH secretion in acromegaly (19, 20). Dopamine receptor D2 is the predominant dopamine receptor found in these adenomas (21, 22), and until the ‘80s dopamine agonists (DA) were the only pharmacological agents for acromegaly treatment. Cabergoline is the DA currently used due to its higher efficacy and better tolerability in comparison to bromocriptine (23, 24). It presents a very safe profile with mild side-effects, is cheap, and is taken orally (25). However, its efficacy, as monotherapy for acromegaly, is relatively low regarding IGF-1 levels reduction; it can be considered as first-line medical therapy only for patients with modestly elevated GH and IGF-1 levels (IGF-1 <2.5 x ULN).

Somatotropinomas express somatostatin receptors (SSTs), especially SST2 and SST5 (26). Somatostatin, as a physiological inhibitor of GH secretion, has been used for the treatment of acromegaly, by developing long-acting compounds. The first-generation short-acting somatostatin receptor ligands (SRLs) octreotide and lanreotide were the first developed (27, 28). Both show a high affinity for SST2 receptors. Thereafter, two long-acting formulations of both octreotide (octreotide Long-Acting Release-LAR) and lanreotide (lanreotide autogel-ATG) were available, allowing monthly injections, a very convenient characteristic for the patient’s compliance. Besides reducing GH and IGF-1, they also provide a substantial tumor reduction effect that makes them very interesting for acromegaly treatment (29–32). Additionally, a new formulation of SRLs, which is allow oral administration of octreotide, has recently demonstrated its efficacy and safety in patient optimally controlled with the parenteral formulation, thus facilitating treatment compliance in those patients reluctant to receive monthly injections (33)

First-generation SRLs have been considered as the first-line medical acromegaly treatment (17) until now. They present a better performance in normalizing GH and IGF-1 levels than cabergoline, although roughly, only about 50% of patients normalize hormonal parameters (34, 35), and large differences in biochemical response rates of first-generation SRLs have been reported (ranging between 25% - 70%). This is probably due in part to different definitions of what is considered as a good biochemical response (36), but certainly also a consequence of the intrinsic heterogeneity of somatotropinomas regarding SSTs expression and other target molecules deployed by these tumors which influence medical therapeutic response. The criteria to define a full response to first-generation SRLs are generally similar across all studies, although with some variations in GH threshold levels. Ideally, both, IGF-1 and GH levels, should be considered for achieving a complete therapeutic response. On the other hand, some authors combine the biochemical targets with the antitumoral effects in the definition of response to first-generation SRLs, but the majority of articles lack a clear cut-off when using these criteria (35, 37).

Moreover, some authors have defined the concept of partial response to SRLs. This latter condition tries to reflect a clinical reality that has led to the implementation of the use of SRLs in combination with other drugs if first-generation SRLs’ effect is somehow clinically significant but doesn’t fully normalize GH and IGF-1 levels.

One of the most used classifications is the one proposed by Colao et al. (36), which defines the full response to SRLs as control of GH and IGF-1 levels together with >20% tumor shrinkage in patients treated as first-line, or control of GH and IGF-1 levels and >20% tumor shrinkage or stabilization of tumor remnant in patients treated in second-line, or no tumor on magnetic resonance imaging at baseline. Partial response includes a significant decrease (>50%) of GH and/or IGF-1 levels with no achievement of normal levels and/or >20% tumor shrinkage. And finally, poor response or resistance to SRLs is defined as a non-clinically significant decrease of GH and IGF-1 levels and no tumor shrinkage.

To avoid the variability over time of IGF-1 measurement, other authors use IGF-1 SD score (SDS). In this case, controlled disease or full response is considered when IGF-1 values are below 2 SDS -or normal by definition-, partial response if SDS is between 2 and 3 SDS, and non-response when greater than 3 SDS (12). First-generation SRLs have been recommended as first-line therapy in non-resectable GH-producing tumors, even if it is well known that they provide biochemical control only in about 50% of cases. Therefore, identifying biomarkers of first-generation SRLs response could be very useful, and nowadays it is still a somehow unmet need. Several studies have proven that surgical debulking of these tumors improves SRLs response (38–40). Consequently, the current general consensus is to perform surgical debulking even if the surgical cure is unlikely, both to alleviate mass effect and to improve SRLs treatment response. Improvement of first-generation SRLs response after surgical debulking seems to be mostly related to the reduction in tumor size, but not all tumors show the same response to SRLs after surgery, even with a similar residual tumor mass.

First-generation SRLs are also used as preoperative treatment for ameliorating comorbidities and reducing tumor volume to improve surgical outcomes (41). A recent meta-analysis has demonstrated better short-term cure rates in acromegaly patients after presurgical SRLs treatment, but its impact on the long-term results is unclear (42).

Pegvisomant was generated by John Kopchick and Wen Chen at Ohio University in 1987 and approved for the treatment of acromegaly in 2003 (43, 44). This GHR antagonist was PEGylated, extending the half-life to about 70 hours. Nowadays, it is used as a second-line treatment for patients not controlled with first-generation SRLs (17).

The first initial trials demonstrated over 90% of IGF-1 normalization in patients resistant to first-generation SRLs (45, 46). Virtually all patients with acromegaly can be controlled with pegvisomant, but clinical, real-life registries showed lower IGF-1 remission rates of about 60-70% (46–48). Pegvisomant rapidly decreases IGF-1 levels in serum and raises GH levels due to the hypothalamic feedback loop (49). Therefore, the only biochemical marker of pegvisomant performance is IGF-1.

Pasireotide and pasireotide-LAR were developed as a multireceptor-targeted SRL with a superior efficacy over octreotide-LAR and it has been considered so far as a second-generation SRLs (50, 51). It could be mostly beneficial in young patients who show tumor growth while receiving medical therapy, in patients with a headache not responsive or intolerant to the other medical treatments and one of the most important group for monotherapy with pasireotide are patients without diabetes which use low pegvisomant doses (≤80 mg/week) during first-generation SRL and pegvisomant combination treatment. Pasireotide may be also used in combination with pegvisomant if the other combination therapies do not control biochemical targets or disease symptoms (52).

In those patients requiring combination treatment with first-generation SRLs and pegvisomant, switching first-generation SRLs to pasireotide in the combined modality is able to reduce by 66% the dose of pegvisomant (53, 54). Of particular interest are the effects of pasireotide upon tumor volume reduction; short-acting pasireotide has shown tumor reduction >20% in 56% of patients after 6 months of treatment (55), and when comparing this effect with the one obtained by octreotide LAR, the mean decrease for this latter compound is 42% volume reduction and 54% for pasireotide (56). In special etiologies of acromegaly, such as in cases of AIP mutation and x-linked acrogigantism in which these patients are bearing large tumors, the indication of pasireotide may be more convenient (57–59).

Pasireotide-LAR shows a good tolerability profile, similar to SRLs. Amelioration of acromegaly symptom score have been reported to be superior with pasireotide than with first-generation SRLs (51). However, hyperglycemia is a relatively common effect and has been perceived as one of the unfavorable factors for its use in acromegaly and Cushing disease, especially in diabetic or prediabetic patients at baseline. However, achieving good glycemic control has been demonstrated manageable with standard anti-diabetic therapy (60, 61).

## Imaging Markers of Response to Pasireotide

The T2-weighted MRI signal has demonstrated to anticipate somatotropinoma response to first-generation SRLs as well as to pasireotide (12, 62). Densely granulated (DG) somatotropinomas use to present a hypointense T2-weighted signal, while most of the somatotropinomas depicting a sparsely granulated pattern use to be either isointense or hyperintense when compared to the cerebral cortex signal. DG pattern is linked to a favorable response to SRLs while SG is not. Moreover, the MRI signal as a predictor of response to first-generation SRLs is also useful after surgical failure and should always be assessed as a response biomarker because surgery does not modify MRI tumor intrinsic signal. Patients showing a hypointense T2-weighted signal use to present a higher percentage of IGF-I decrease after 6 months of treatment with first-generation SRLs; and conversely there is a higher percentage of patients with hyperintense signal achieving less than 50% decrease in IGF-I (63). As MRI is always done in patient diagnosis assessment, no matter if the surgical treatment will be performed or not, the inclusion of T2-weighted signal evaluation is very recommendable as it helps to identify patient response to treatment. Finally, machine learning-based texture analysis of T2-weighted MRI images has recently been developed and can correctly classify response to first-generation SRLs in more than 80% of the patients. Machine learning-based texture analysis performs better than qualitative and quantitative evaluation of relative T2 signal intensity and immunohistochemical evaluation (64).

T2-weighted relative signal intensity (rSI) has been also strongly correlated with biochemical sensitivity to SRLs. The cut-off value of T2-weighted rSI to distinguish biochemical sensitivity was found in one study to be 1.205, with a positive predictive value (PPV) of 81.5% and a negative predictive value (NPV) of 77.3% (65). Inasmuch, T2-weighted rSI correlated with the expression of SST5 and quantitatively predicted the biochemical efficacy of first-generation SRLs. These findings are of much interest regarding the prediction of response to pasireotide, thus further studies in this regard are warranted.

T2-weighted hyperintensity signal has been recently linked to the identification of responsiveness to pasireotide (62), since higher T2-signal intensity adenomas at baseline were correlated with better hormonal response levels during 3 and 9 months of pasireotide treatment and not tumor shrinkage. But shrinkage is also frequently observed in tumors harboring hyperintense T2-weighted MRI signal when treated with this compound, supporting the potential antitumor activity of pasireotide (66).

## Functional Tests for Prediction of Pasireotide Response

Functional tests may help to identify either pathologic hormonal situations as well as response to specific compounds; this may also be the case for pasireotide. The acute octreotide test (AOT) was initially formulated in 1989 by Lamberts in order to evaluate the duration of the effect of short-acting somatostatin therapeutic formulations available in the late ‘80s and thus deciding the number of injections per day required for a given patient. Since the refinement of SRLs preparations, with long-acting compounds available for more than 15 years, the AOT was virtually abandoned until this procedure was reconsidered as a potentially useful tool for prediction of response to long-acting SRLs. Some studies evaluating its performance as a predictor of therapeutic response have been published, being the overall results somehow conflictive, because not all the studies concluded that the AOT was sufficiently predictive of a good or a bad response to first-generation SRL. The methodology used and the definition of nadir was quite heterogeneous between the studies. As a consequence, some clinicians have decided not to use it (67–69), while others are in favor of using it (13, 14, 70–72).

We formulated a short version of the AOT that was able to predict quite accurately the long-term response to SRLs treatment in acromegalic patients. Mostly, it serves to identify non-responders to first-generation SRLs, thus allowing progress in the classic sequential therapeutic algorithm and introducing the next available drugs, mostly until recently, pegvisomant or cabergoline in those cases of almost complete response to SRLs. In our experience, a GH nadir of 9.2 ng/ml predicted IGF1 <3 SD with 82% sensitivity and 58% specificity (75% PPV, 67% NPV), and a GH nadir 3.6 ng/ml predicted IGF1<2 SD with a 75% sensitivity and a 58% specificity (33% PPV and 89% NPV); therefore, the NPV clearly serves to distinguish between partial non-responders and true SRLs resistant cases. This short version test is cost-effective and useful in the clinical practice as with two GH measurements -basal and at 2 hours nadir after sc injection of 100 mcg of regular somatostatin-, it is sufficient for response assessment. In our initial series in which 26 consecutive patients were studied, we found a positive correlation between post-treatment IGF-I values at 12 months and GH levels during AOT (r_s_ 0.76; p>0.0001). The AOT may be also useful after surgical debulking, as a decreased residual tumor may become responsive to SRLs in some patients (38–40) and this new situation could be identified with a postsurgical AOT.

In a more recent study by Wang et at in 2016, using the long 6-hour version it was found that the AOT was efficient and accurate for identification of SRLs response (sensitivity 93.8%, specificity 85.7%); AOT was also very informative regarding tumor size decrease prediction (sensitivity 84.8%, specificity 87.5%), as well as providing definite and consistent data on the usefulness of such procedure (14).

In the light of this data, it is evident that a pasireotide acute test should be designed and its value assessed as a predictor to pasireotide LAR response in acromegaly. It may be relatively feasible to perform a validation study at an international level in which this hypothesis would be proved. Performing an acute octreotide and a pasireotide test in a single patient in a 24 h period would allow to know very quickly which treatment would work best for a given patient, saving time to achieve hormonal control.

## Molecular Markers of Response to Pasireotide

As for the treatment of acromegaly for patients not cured by surgery, different studies have been performed so far in order to identify biomarkers of response to SRLs in these cases, which accounts for about 70% of the cases in which invasion of the cavernous sinus is present. The most recent information regarding this issue indicates that E-cadherin as well as *SST2* are the best predictors of response to these drugs (16, 73), although other molecules may be involved in the complex post-SSTs pathway and this includes RAF-kinase, MAPK, *PLAGL1*-*AIP*, RET/Pit1/p14ARF (74) pathways, as well as Ki-67 (75), EMT markers (76, 77) and the presence of truncated SSTs (78).

Regarding pasireotide, due to its SSTs unique multi-ligand capacity, it is expected that either SST2 or SST5 quantitative expression measurements may inform to what extent those tumors depicting these SSTs might be more prone to a positive therapeutic response. Pasireotide shows the highest affinity for SST5, followed by SST2, SST3, and SST1 (79), although it has a slightly lower affinity -but still important- to SST2 than first-generation SRLs. In recent studies (15, 80, 81), it has been shown that in somatotropinomas depicting a SST5 immunohistochemical score of 2 or 3, a positive response to pasireotide is observed in about 50% of the cases resistant to first-generation SRLs; SST3 and AIP expression did not influence the response to pasireotide, while sparsely granulated adenomas responded better compared to densely granulated. However, in most of the tumors of unselected patients, pasireotide seems to exert its biological effects predominantly through SST2 (82). Moreover, a lack or a very low SST5 expression is in any case a strong predictor of non-responsiveness to pasireotide (15). And, in fact, even a low expression of SST2 is sufficient to activate a biologic response as pasireotide does not stimulate SST2 internalization, as first-generaton SRLs do, and therefore the receptor remains longer in the membrane (83). Only in those subjects resistant to first-generation SRLs, harboring tumors with negligible SST2 expression, pasireotide can be also effective acting particularly through SST5 (80). Besides the different receptor binding affinity, pasireotide exhibits different functional properties compared to first-generation SRLs when binding to SST5, and particularly SST2. However, in one of the recently published studies, high expression of SST5 was controversially associated with pasireotide resistance in some cases (81). Also, AIP low expression tumors may be pasireotide-responsive, indicating that this pathway is not exclusively implicated in the biological response to pasireotide, in opposition to what happens with first-generation SRLs, in which it plays an important role; this has been recently described in AIP mutated acromegaly patients (84). Additionally, data from a somatotroph-specific AIP knock-out mouse model have shown a lowering IGF-1 effect of pasireotide (85). Hence, it may be proposed that the best pharmacologic treatment in acromegaly AIP mutated patients should be pasireotide.

Pasireotide is able to activate phosphorylation of Threonine 333 residue of SST5, a key specific biological target of pasireotide action regulating SST5 trafficking and internalization, while this is not observed for first-generation SRLs (86).These differences include SSTs pathway activation and modulation of receptors phosphorylation, internalization, and trafficking, involving a number of molecules that regulate membrane receptor functions, such as β-arrestins (87).

Taken together, these data indicate that, in addition to effectiveness conferred through SST2 binding (74), those tumors with a specific biologic profile, namely low or absent SST2 and high SST5 in a low AIP expression sparsely granulated rather than densely granulated, will also be responsive to pasireotide and in these particular cases, it would be the best pharmacologic treatment option. However, if a higher accuracy is needed in single patients’ prediction when it comes to first-generation SRLs, the same may apply for prediction of response to pasireotide; thus, validation studies are warranted for this latter.

GH receptor (GHR) polymorphisms, and particularly d3-GHR have been involved in different sensitivity to GH and to the different necessity of pegvisomant dosage to get hormonal control during acromegaly treatment (88). Surprisingly, d3-GHR has been linked to resistance to pasireotide treatment in a retrospective study (81). Although no explanation is so far available, a worse response to first-generation SRLs was described in patients carrying d3-GHR genotype (89).

Therefore, although current results are very promising, additional research in the determinants of pasireotide response is required for the identification of more biomarkers able to complement current predictive factors and to increase the required accuracy for personalized treatment.

## Discussion

The “trial and error” approach that has driven clinical practice of acromegaly patients until now, based on the sequential addition of different compounds starting always with SRLs, is no more sustainable in the 21st century considering the high failure rate of these latter drugs. The delay in controlling the disease in patients who do not respond to SRLs as the first-line treatment can be quantified in years, as every modification in the medical therapy requires some months to be fully evaluated. Thus, it has been proposed that acromegaly patients should benefit from a therapeutic approach by using molecular analysis but also the information provided by functional and imaging procedures (7–9, 90, 91).

**Figure 1:**
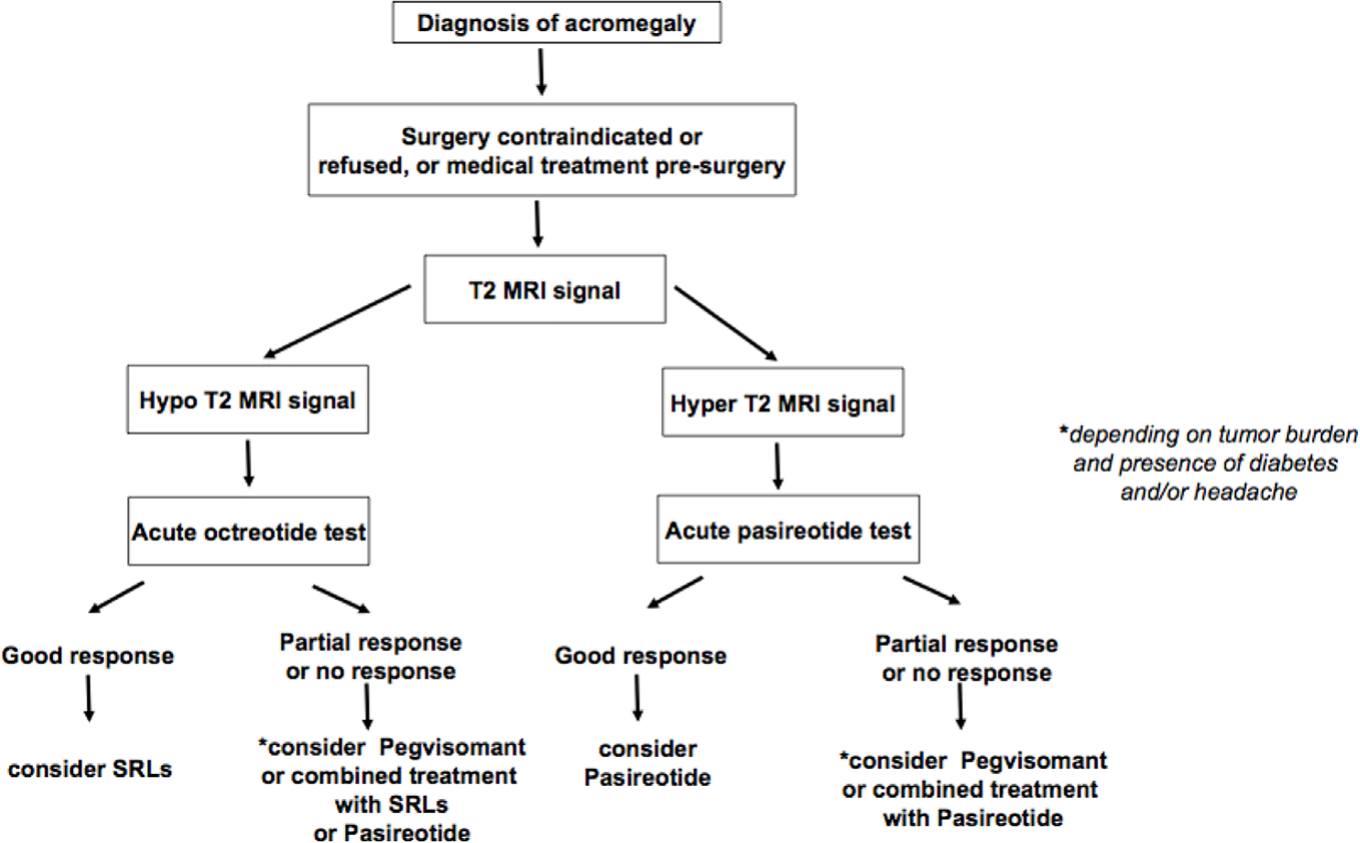
Therapeutic algorithm for medical treatment of acromegaly before surgery.

Thus, a modern treatment algorithm of acromegaly should be based on the selection of patients at the time of diagnosis according to their T2 MRI signal and response to regular octreotide and pasireotide acute tests, provided that the latter will be developed and validated. Those patients with a positive response to the acute compound tests would be good candidates for any of them, and a specific choice would also consider clinical conditions particularities such as age and diabetes coexistence, as well as the presence of headache. Together with this, hypointensity T2-weighted signal would support the use of first-generation SRLs while hyperintensity would support pasireotide. In those cases in which acute octreotide or pasireotide test would not achieve predictive cut-off of full response, or imaging is not concordant, the addition of pegvisomant as a combination or if cut-off of the acute test indicates resistance to first-generation SRLs or pasireotide, monotherapy with pegvisomant would be the best option (**Figure 1**).

**Figure 2:**
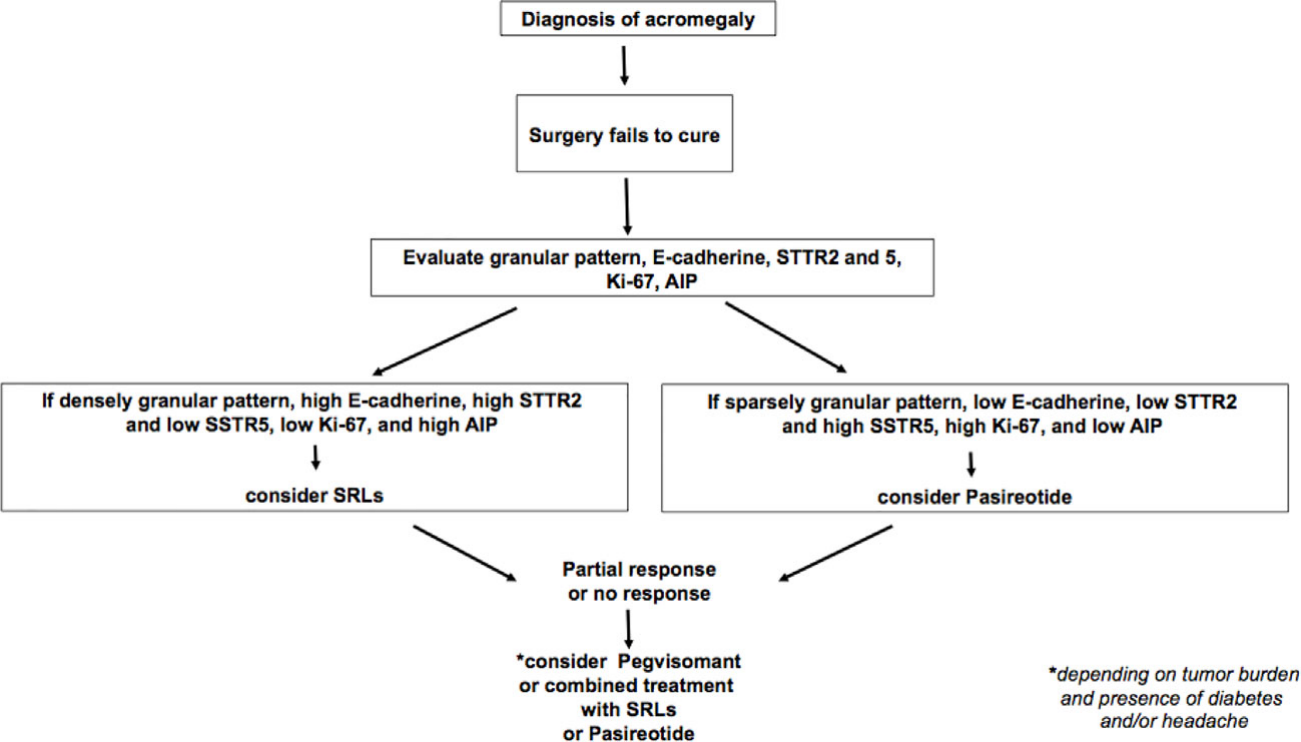
Therapeutic algorithm for medical treatment of acromegaly after non-curable surgery.

After surgical therapy, consideration of molecular and pathologic patterns may be very helpful in those cases not achieving curation. Therefore, when the densely granulated pattern and high expression of E-cadherin, SST2, low SST5, and Ki-67 are present, first-generation SRLs would be the best option, while the opposite pattern, namely, sparsely granulated pattern, low SST2 and E-cadherin, detectable SST5, low or absent *AIP*, and high Ki-67, would best fit for pasireotide (**Figure 2**). As pegvisomant does not act on the tumor itself, this latter information does not apply in the decision-making process to implement this compound, and obviously, the best clinical practice judgement from an experienced team would prevail when conflicting information is present in a given patient.

The current scientific knowledge is starting to be consistent enough to change the spirit of “trial and error” therapeutic algorithm and promote a much modern one that would allow precision medicine soon. Some tools are still waiting to be developed, such as the acute pasireotide test, that certainly will be of much aid in the context of personalized medicine.

## Author Contributions

MP-D, IB, MM and BB contributed to conception and design of the review. MP-D wrote the main manuscript. All authors contributed to the article and approved the submitted version.

## Funding

This work was partly supported by a grant from Instituto Carlos III, Madrid, Spain, on personalized treatment of acromegaly (PMP:15/00027). Recordati financed medical writing for style and editing but did not influence nor had access to the content of this paper before its submission.

## Conflict of Interest

MP-D, IB, CA-E, AP, M-AG, RC, BB, JA, CL, and MM received grants support and participated in speaker bureau for Pfizer, Recordati, Ipsen, and Novartis.

The remaining authors declare that the research was conducted in the absence of any commercial or financial relationships that could be construed as a potential conflict of interest.
